# Phenotypic and Genotypic Antimicrobial Resistance Traits of *Vibrio cholerae* Non-O1/Non-O139 Isolated From a Large Austrian Lake Frequently Associated With Cases of Human Infection

**DOI:** 10.3389/fmicb.2019.02600

**Published:** 2019-11-08

**Authors:** Sarah Lepuschitz, Sandrine Baron, Emeline Larvor, Sophie A. Granier, Carina Pretzer, Robert L. Mach, Andreas H. Farnleitner, Werner Ruppitsch, Sonja Pleininger, Alexander Indra, Alexander K. T. Kirschner

**Affiliations:** ^1^Austrian Agency for Health and Food Safety (AGES), Institute for Medical Microbiology and Hygiene, Vienna, Austria; ^2^Research Division of Biochemical Technology, Institute of Chemical, Environmental and BioScience Engineering, Technische Universität Wien, Vienna, Austria; ^3^French Agency for Food, Environmental and Occupational Health & Safety (ANSES), Ploufragan-Plouzané-Niort Laboratory, Ploufragan, France; ^4^French Agency for Food, Environmental and Occupational Health & Safety (ANSES), Fougeres Laboratory, Fougeres, France; ^5^Institute for Hygiene and Applied Immunology - Water Microbiology, Medical University Vienna, Vienna, Austria; ^6^Division Water Quality and Health, Karl Landsteiner University of Health Sciences, Krems an der Donau, Austria; ^7^Interuniversity Cooperation Centre for Water and Health, Vienna, Austria

**Keywords:** *Vibrio cholerae*, antibiotic resistance, bathing water, climate change, non-O1/non-O139

## Abstract

*Vibrio cholerae* belonging to serogroups other than O1 and O139 are opportunistic pathogens which cause infections with a variety of clinical symptoms. Due to the increasing number of *V. cholerae* non-O1/non-O139 infections in association with recreational waters in the past two decades, they have received increasing attention in recent literature and by public health authorities. Since the treatment of choice is the administration of antibiotics, we investigated the distribution of antimicrobial resistance properties in a *V. cholerae* non-O1/non-O139 population in a large Austrian lake intensively used for recreation and in epidemiologically linked clinical isolates. In total, 82 environmental isolates - selected on the basis of comprehensive phylogenetic information - and nine clinical isolates were analyzed for their phenotypic antimicrobial susceptibility. The genomes of 46 environmental and eight clinical strains were screened for known genetic antimicrobial resistance traits in CARD and ResFinder databases. In general, antimicrobial susceptibility of the investigated *V. cholerae* population was high. The environmental strains were susceptible against most of the 16 tested antibiotics, except sulfonamides (97.5% resistant strains), streptomycin (39% resistant) and ampicillin (20.7% resistant). Clinical isolates partly showed additional resistance to amoxicillin-clavulanic acid. Genome analysis showed that *crp*, a regulator of multidrug efflux genes, and the bicyclomycin/multidrug efflux system of *V. cholerae* were present in all isolates. Nine isolates additionally carried variants of *bla*_CARB–7_ and *bla*_CARB–9_, determinants of beta-lactam resistance and six isolates carried *catB9*, a determinant of phenicol resistance. Three isolates had both *bla*_CARB–7_ and *catB9*. In 27 isolates, five out of six subfamilies of the MATE-family were present. For all isolates no genes conferring resistance to aminoglycosides, macrolides and sulfonamides were detected. The apparent lack of either known antimicrobial resistance traits or mobile genetic elements indicates that in cholera non-epidemic regions of the world, *V. cholerae* non-O1/non-O139 play a minor role as a reservoir of resistance in the environment. The discrepancies between the phenotypic and genome-based antimicrobial resistance assessment show that for *V. cholerae* non-O1/non-O139, resistance databases are currently inappropriate for an assessment of antimicrobial resistance. Continuous collection of both data over time may solve such discrepancies between genotype and phenotype in the future.

## Introduction

Antibiotics are supposed to be one of the most effective drugs to treat infectious diseases transmitted by bacteria (Martinez, 2009). Nevertheless, antimicrobial resistant bacteria pose one of the most significant healthcare challenges (WHO, 2017). Emerging antibiotic resistance is not only restricted to healthcare associated settings, but also appears in environmental settings, such as natural aquatic ecosystems (Taylor et al., 2011). The Gram-negative, potentially pathogenic bacterium *Vibrio cholerae* is a natural inhabitant of aquatic environments from freshwater to seawater. It can thrive in a free floating manner in the water body (Worden et al., 2006; Kirschner et al., 2008) but it has also been found on surfaces of crustaceans, algae, plants, and insects (Colwell, 1996; Halpern et al., 2004; Banerjee et al., 2014). Currently, more than 200 different serogroups have been described and only serogroups O1 and O139 are the agents of epidemic cholera. The remaining non-epidemic serogroups are therefore referred as *V. cholerae* non-O1/non-O139. They are opportunistic pathogens associated with small outbreaks of diarrheal disease (Deshayes et al., 2015; Hasan et al., 2015; Crowe et al., 2016). They can also cause severe wound infections and acute sepsis especially in immunocompromised patients (Sack et al., 2004; Huhulescu et al., 2007; Hirk et al., 2016; Siriphap et al., 2017). In recent years, infections due to *V. cholerae* non-O1/non-O139 have been increasingly reported for countries of the Northern hemisphere (Newton et al., 2012; Baker-Austin et al., 2013), primarily acquired through contact by swimming in seawater (Baker-Austin et al., 2016b; Marinello et al., 2017) or freshwater lakes (Dalsgaard et al., 2000a; Ninin et al., 2000; Andersson and Ekdahl, 2006; Ottaviani et al., 2011; Dobrovic et al., 2016; Hirk et al., 2016; Kechker et al., 2017). This significant increase in the number of infections has been linked to global warming and associated increase in water temperatures (Baker-Austin et al., 2016a, b; ECDC, 2019). Infections caused by *V. cholerae* non-O1/non-O139 have also been reported for the large subsaline lake Neusiedler See in Austria (Huhulescu et al., 2007). This lake is part of the largest bird sanctuary in Central Europe (National Park Neusiedler See - Seewinkel) and it is intensively used for recreational purposes throughout the year (swimming, surfing, sailing, fishing etc.). Recently, it was demonstrated, that the lake harbors an abundant and genetically diverse endemic *V. cholerae* community (Schauer et al., 2015; Pretzer et al., 2017). In the latter study (Pretzer et al., 2017), it was also shown that many *V. cholerae* isolates from other European countries were genetically related to the strains present in the lake, most probably due to the long distance import of strains (via birds or humans) from these countries. Thus, the Neusiedler See may serve as a bioreactor for the appearance of new strains with new (pathogenic) properties, including antibiotic resistance.

Despite the increasing clinical importance of environmental *V. cholerae*, only very limited information is available on antimicrobial susceptibility of *V. cholerae* non-O1/non-O139 populations in aquatic ecosystems of the Northern hemisphere (Aberkane et al., 2015; Bier et al., 2015; Baron et al., 2017). Drug resistance of *V. cholerae* is mediated by efflux pumps, by chromosomal mutations and by acquisition of conjugative plasmids, transposons, integrative and conjugative elements (ICE) such as SXT elements carrying antibiotic resistance genes (Kitaoka et al., 2011; Das et al., 2019). Aquatic ecosystems provide a suitable milieu for spreading antimicrobial resistant traits through horizontal gene transfer among bacterial populations (Baquero et al., 2008). Recent publications reported a limited capacity of *V. cholerae* non-O1/non-O139 strains to acquire resistance. Apparently, most of the investigated collections from Western European waters were susceptible to most antibiotic tested (Bier et al., 2015; Baron et al., 2017). However, multidrug resistant *V. cholerae* have been increasingly reported worldwide, mainly in clinical O1 and O139 strains (Faruque et al., 2007; Mandal et al., 2011; Verma et al., 2019), but also in environmental non-O1/non-O139 isolates in cholera epidemic areas (Kitaoka et al., 2011). First carbapenemase producing *V. cholerae* non-O1/non-O139 strains were recently described in Western Europe (Aberkane et al., 2015; Bier et al., 2015). Therefore, monitoring antimicrobial resistance of environmental populations of *V. cholerae* non-O1/non-O139 is highly relevant for public health.

The aim of this study was to determine the phenotypic and genotypic antimicrobial resistance properties of 91 *V. cholerae* non-O1/non-O139 strains (82 representative isolates from Neusiedler See and 9 Austrian clinical, epidemiologically linked isolates), by combining *in vitro* antimicrobial susceptibility testing and *in silico* detection of antimicrobial resistance traits from whole genome sequencing (WGS) data. By this a comprehensive assessment of the role of the *V. cholerae* population in Neusiedler See as a potential reservoir of environmental antimicrobial resistance was achieved. Since this lake may serve as model bioreactor for the appearance of new pathogenic/resistant *Vibrio* strains our findings are also relevant for other subsaline/hyposaline aquatic environments that are used for recreation.

## MATERIALS AND METHODS

### Description of the Study Area and Origin of Isolates

Neusiedler See is a large (320 km^2^) subsaline, alkaline lake in Eastern Austria, intensively used for recreational purposes with several hundred thousand visitors throughout the year. The lake is located in the temperate climate zone and has experienced an increase in the average water temperature during the past 30 years of around 1.5°C (Dokulil et al., 2010). Details on the lake can be found in earlier publications (Kirschner et al., 2008; Schauer et al., 2015).

Eighty-two environmental *V. cholerae* non-O1/non-O139 isolates for the analysis of antibiotic resistance properties were selected from an existing strain collection consisting of 472 isolates (Pretzer et al., 2017). The majority of these 472 isolates belong to 24 phylogenetic clades, based on multi-locus sequence analysis (MLSA) (Pretzer et al., 2017). Depending on the size of the clades (i.e., the number of isolates belonging to the clades), between one and six representatives of each clade (*n* = 46) and additional singletons (*n* = 36) were randomly selected, in order to cover the bulk of the genetic diversity of *V. cholerae* in the lake (Supplementary Figure S1). All selected *V. cholerae* environmental isolates were collected at Neusiedler See between May 2011 and October 2012 (Supplementary Figure S1).

For comparison, nine *V. cholerae* non-O1/non-O139 strains from human infections which had a reported epidemiological link to Neusiedler See, collected between 2000 and 2015, were also included in this study (Supplementary Table S1). All of these strains had been sent to the national reference laboratory for *Vibrio* at the Austrian Agency for Health and Food Safety (AGES). Six of the strains had caused otitis externa, one of them sepsis, another one prostatitis and for one strain no clinical symptoms had been reported.

### Phenotypic Susceptibility Testing

The susceptibility of the 91 isolates was determined by disk diffusion method according to the Clinical and Laboratory Standards Institute (CLSI) guidelines for rapidly growing pathogens (CLSI, 2018b) including the following 16 antimicrobial agents: ampicillin, amoxicillin-clavulanic acid, cefotaxime, imipenem, chloramphenicol, nalidixic acid, ciprofloxacin, norfloxacin, amikacin, gentamicin, streptomycin, tetracycline, doxycycline, sulfonamides (sulfamethoxazole, sulfisoxazole, sulfadiazine), trimethoprim-sulfamethoxazole, and erythromycin. The CLSI interpretative criteria for disk diffusion susceptibility testing of Vibrio spp. (CLSI, 2015, 2018a) were used (Supplementary Table S2), except for erythromycin for which no breakpoint was available. In Baron et al. (2017), the disc diffusion zones of inhibition of the wild type population were distributed between 17 and 24 mm. Therefore, any erythromycin inhibition zone diameter measured larger or equal to 17 mm was interpreted as susceptible. Non-susceptible categories (intermediate and resistant) were interpreted as resistant for this study. The term “multidrug resistant” was used for isolates being non-susceptible to at least one agent of three or more different antimicrobial classes as previously defined by ECDC and CDC experts (Magiorakos et al., 2012).

### Whole-Genome Sequencing and Antimicrobial Resistance Gene Detection

From the 82 phenotypically tested environmental isolates, 46 isolates were selected for WGS from all but three identified phylogenetic clades (*n* = 36) and a few singletons (*n* = 10), in order to cover the bulk of the genetic diversity of *V. cholerae* in Neusiedler See (Supplementary Figure S1). In addition, eight of the nine clinical isolates were also sequenced. Bacterial total DNA was isolated from overnight cultures using the MagAttract HMW DNA Kit (Qiagen, Hilden, Germany) and DNA concentration was determined with a Trinean DropSense16 (Unchained Labs, CA, United States). Libraries for WGS were prepared using the Nextera XT DNA Library Preparation Kit (Illumina, CA, United States) according to the manufacturer’s instructions. All isolates were paired-end sequenced using MiSeq Reagent Kit v3 with a read length of 2 × 300 base pairs on a MiSeq instrument (Illumina, CA, United States). Velvet version 1.1.04 (Zerbino and Birney, 2008) was used for *de novo* assembly of raw reads and further WGS data interpretation was carried out in the analysis software SeqSphere + (Ridom, Münster, Germany).

The presence of antibiotic resistance genes was assessed with the Comprehensive Antibiotic Resistance Database (CARD) (Jia et al., 2017) using default settings including “Perfect” and “Strict” hits only. In addition, the ResFinder database was used with default settings. Allele libraries for aminoglycosides (167 targets), macrolides (132 targets) and sulfonamides (49 targets) resistance were downloaded from the ResFinder database (11/2017) available from the Center for Genomic Epidemiology^1^ and integrated in the SeqSphere + analysis software. *In silico* target scan procedure settings in SeqSphere + for reference sequences were defined with 90% required identity to reference sequence and 99% aligned to reference sequence. The following reference sequences were included: species-specific gene (*ompW;* accession no. FJ462451), SXT element integrase gene (*int;* AF099172), class 1 integron (*intI1;* EU436855), dihydrofolate reductase (*dfrA18;* AY034138), quinolone resistance protein (*qnrVC1;* EU436855), bicyclomycin/multidrug efflux system from *V. cholerae* O1 biovar El Tor str. N16961 (locus VC1634) and *VcmA*, *VcmB*, *VcmD*, *VcmH*, *VcmN*, *VcrM* belonging to the MATE family multidrug efflux pumps (Multidrug And Toxic Compound Extrusion) (Huda et al., 2001, 2003; Begum et al., 2005; Kuroda and Tsuchiya, 2009).

### Nucleotide Sequence Accession Numbers

This Whole Genome Shotgun project has been deposited at DDBJ/EMBL/GenBank under the accession PRJNA551929^2^. For details see Supplementary Table S3. The version described in this paper is the first version.

## Results

### Phenotypic Susceptibility

#### Environmental Isolates

**FIGURE 1:**
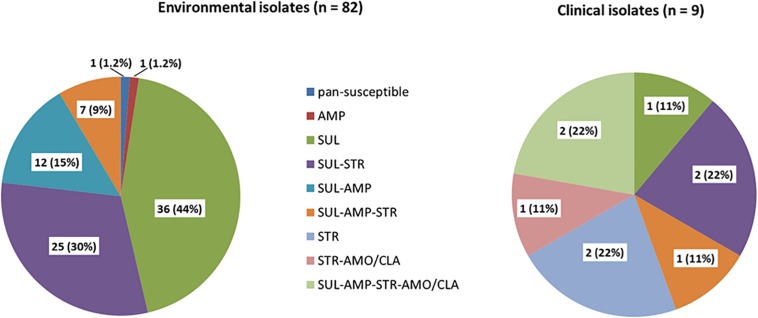
Percentage of phenotypic antibiotic resistance of 82 environmental and nine clinical isolates. SUL (sulfonamides), STR (streptomycin), AMP (ampicillin), NAL (nalidixic acid), AMO/CLA (amoxicillin-clavulanic acid).

All isolates were susceptible to amoxicillin-clavulanic acid, cefotaxime, imipenem, chloramphenicol, ciprofloxacin, norfloxacin, amikacin, gentamicin, tetracycline, trimethoprim, trimethoprim-sulfamethoxazole (SXT) and erythromycin. All but one of 82 isolates were resistant to at least one of the tested antibiotics (Figure 1). Eighty (97.5%) were resistant to sulfonamides, 54 of them were only detected resistant to this antibiotic (44%). Twenty-five isolates (30%) were resistant to sulfonamides and streptomycin, twelve (15%) to sulfonamides and ampicillin and one isolate (1.1%) was only resistant to ampicillin. Seven isolates (9%) were multidrug resistant against sulfonamides, streptomycin and ampicillin.

#### Clinical Isolates

Overall, antibiotic resistance profiles were very similar in human and environmental isolates. Resistances against sulfonamides, streptomycin and ampicillin were the most frequently detected (Figure 1). The main difference observed was the occurrence of amoxicillin/clavulanic acid resistance in three clinical isolates. Three out of the nine isolates were multidrug resistant. None was pan-susceptible.

The full detailed list of all isolates tested and the results of each phenotypic antimicrobial resistance test can be found in the Supplementary Table S4.

### Identification of Antibiotic Resistance Genes

The coverage obtained for the 54 whole genome sequenced isolates (46 environmental and eight clinical) was 68.2-fold and the species-specific gene *ompW* for *V. cholerae* was present in all investigated isolates. Analysis via CARD identified gene *tet*(34), an antibiotic inactivation enzyme and determinant of tetracycline resistance, and gene *CRP*, a regulator of multidrug efflux genes, to be present in all 54 investigated isolates (Supplementary Table S4).

Twelve isolates additionally carried variants of the antibiotic inactivation enzymes *bla*_CARB–7_ (*n* = 10) or *bla*_CARB–9_ (*n* = 2), determinants of beta-lactam resistance, perfectly matching the observed phenotypic resistance against the beta-lactam antibiotic ampicillin. Six isolates additionally carried the inactivation enzyme *catB9*, a determinant of phenicol resistance, despite the fact that no chloramphenicol resistance was observed for these isolates. Three isolates carried both *bla*_CARB–7_ and *catB9*. For all 54 isolates, no genes conferring resistance to aminoglycosides, macrolides nor sulfonamides were detected. The integron 1 integrase gene *intI1*, the two SXT integron-related resistance factors *dfrA18*, *qnrVC1* and diverse SXT elements were absent in all investigated isolates. The bicyclomycin/multidrug efflux system locus of *V. cholerae* strain VC1634, which is involved in sulfonamide and bicyclomycin resistance, was present in all isolates. In 27 of 54 isolates five (*VcmA, VcmB, VcmD, VcmH, VcrM*) out of six subfamilies of the MATE (Multidrug *and toxic* compound extrusion)-family were present (Table 1 and Supplementary Table S4). *VcmN* was never detected.

**TABLE 1:** Phenotypic and genotypic resistance profiles of the 54 whole genome sequenced *V. cholerae* isolates.

**Source**	**Phenotypic resistance**	**Number of**	***tet*(34)**	***catB9***	***bla*_CARB–7_**	***bla*_CARB–9_**	**Bicyclomycin/multidrug**	**MATE multidrug**	***CRP***
	**profile**	**isolates**					**efflux system**	**efflux pump**	
**Environmental**	SUL	1	+	+	−	−	+	+	+
**(*n* = 46)**		17	+	−	−	−	+	+	+
	SUL, AMP	4	+	−	+	−	+	+	+
		2	+	−	−	+	+	+	+
	SUL, STR	4	+	+	−	−	+	+	+
		15	+	−	−	−	+	+	+
	SUL, AMP, STR	1	+	+	+	−	+	+	+
		2	+	−	+	−	+	+	+
**Clinical (*n* = 8)**	STR	2	+	−	−	−	+	+	+
	STR, SUL	1	+	−	−	−	+	+	+
		1	+	+	−	−	+	+	+
	STR, AMO/CLA	1	+	−	−	−	+	+	+
	STR, AMP, SUL	1	+	+	+	−	+	+	+
	STR, AMP, SUL, AMO/CLA	1	+	+	+	−	+	+	+
		1	+	−	+	−	+	+	+

*Phenotypic resistance was determined according to CLSI breakpoints (CLSI, 2015, 2018b): SUL: sulfonamides, AMP: ampicillin, STR: streptomycin; AMO/CLA: amoxicillin/clavulanic acid; the presence of antibiotic resistance genes was extracted from WGS data: ± = gene or mutation was present/absent. Only resistance targets with at least one positive isolate are shown.*

## Discussion

### All Strains Are Susceptible to First-Line Treatment Options and Carbapenems

All environmental and clinical isolates were susceptible to ciprofloxacin, norfloxacin, doxycycline and extended spectrum cephalosporins, the recommended treatment options for *V. cholerae* non-O1/non-O139 infections (Daniels and Shafaie, 2000). Thus, a loss of effectiveness of first-line treatments for *V. cholerae* infections related to Neusiedler See is not observed so far. Moreover, in agreement with the susceptibility to extended spectrum cephalosporins and carbapenems, no extended spectrum β-lactamase or carbapenemase genes were identified. Carbapenemase genes in *Vibrio cholerae* non-O1/non-O139 populations are uncommon in Europe, carbapenemase genes were so far only found in avian swab samples in France (Aberkane et al., 2015), a United Kingdom patient flying back from India (Darley et al., 2012) and the presence of undefined carbapenemases was reported for four Baltic Sea isolates (Bier et al., 2015).

### Wide-Spread Resistance Against “Old Antibiotics”

Among the 82 environmental isolates, phenotypic resistances were only observed against the “old antibiotics” such as sulfonamides, streptomycin and ampicillin which had been widely used before the 1970’s, 9% of the isolates were multidrug resistant. Widespread resistance of *V. cholerae* non-O1/non-O139 strains against these substances has also been observed in wastewater and cockles of a Western European estuary (Baron et al., 2017). In other aquatic ecosystems from cholera free regions of the temperate climate zone, resistance to streptomycin and ampicillin was reported as well in the Baltic Sea and the North Sea (Bier et al., 2015) and the Chesapeake Bay (Ceccarelli et al., 2015). However, resistance to sulfonamides alone was not tested in those studies but only in combination with trimethoprim. Overall, the percentage of resistance against these three antibiotics was higher in Neusiedler See in comparison to the other investigations. In our study, 98% of the strains were resistant to sulfonamides, while in the Western European estuary it was 45%. Concerning ampicillin, 24% of our isolates showed resistance in comparison to 9% (Baron et al., 2017), 10% (Bier et al., 2015) and 7% (Ceccarelli et al., 2015); concerning streptomycin it was 39% resistant isolates from Neusiedler See in comparison to 22% (Baron et al., 2017), 25% (Bier et al., 2015) and 8% (Ceccarelli et al., 2015). The high rates of acquired resistances against sulfonamides and streptomycin in the isolates of Neusiedler See indicate a continuous selective pressure from these antibiotic classes. So far, there is no evidence showing the presence of those antibiotics in the lake triggering such resistance and monitoring of these antibiotics in the lake would be necessary. Currently, the presence of antibiotics in water by constant monitoring is not regulated in Austria and other European countries, which makes it difficult to assess the actual cause for the observed antibiotic resistance of *V. cholerae* non-O1/non-O139 strains (Inreiter et al., 2016). Streptomycin is widely used in plant agriculture, predominantly for combating fire blight and it has been permitted on an emergency use basis, subject to annual review and under tightly restricted conditions, in Austria (Stockwell and Duffy, 2012). High acquired resistance rates to sulfonamides have been observed in *V. cholerae* O1 (Eibach et al., 2016) but investigations on *V. cholerae* non-O1/non-O139 are rare. Concerning resistance to beta-lactams, the first strain of *V. cholerae* with acquired resistance to ampicillin due to a plasmid located beta-lactamase has been detected in 1977 (Hedges et al., 1977). From the 1990s onward several studies describe the prevalence of ampicillin resistance observed in environmental strains of *V. cholerae* non-O1/non-O139 in different parts of the world (Choury et al., 1999; Dalsgaard et al., 2000b; Melano et al., 2002; Petroni et al., 2004). The ampicillin resistance genes are carried by class 1 integrons. At least three carbenicillin-hydrolyzing beta-lactamases (CARB-6, CARB-7, and CARB-9) have been described in *V. cholerae* (Choury et al., 1999; Melano et al., 2002; Petroni et al., 2004). In our study, twenty ampicillin resistant environmental isolates were identified. From the nine sequenced isolates, a perfect match between the presence beta-lactamase genes and ampicillin resistance phenotype was observed.

The clinical isolates showed similar resistance patterns as the environmental isolates. However, three of them (33%) were additionally resistant to the combination of amoxicillin and clavulanic acid. No such resistance was found for patient and environmental isolates from the North and Baltic Sea region (Bier et al., 2015), but 19 of 42 environmental isolates from the Adriatic Sea (45%) and 3 of 9 Italian clinical isolates (33%) were resistant to the combination of penicillins and beta-lactamase inhibitors (Ottaviani et al., 2018). Nevertheless, prudent interpretation of the present data is required, as the three strains categorized as resistant to amoxicillin – clavulanic acid, displayed an inhibition zone diameter just below the susceptibility breakpoint. This breakpoint is supposed to be used “for *Vibrio* spp. other than *V. cholerae*” (CLSI, 2015). Due to limitations of the method, it can be hypothesized that these three strains have probably been falsely categorized as resistant.

### Divergent Phenotypic and Genotypic Antimicrobial Profiles

Several divergent phenotypic and genotypic antimicrobial findings were obtained. All sequenced isolates harbored the *tet*(34) gene, encoding for a tetracycline inactivation enzyme (Nonaka and Suzuki, 2002), while all isolates were susceptible to tetracyclines. It should be noticed that the *tet*(34) gene was originally cloned from a *Vibrio* spp. and oxytetracycline resistance was only expressed in presence of MgCl_2_ (Nonaka and Suzuki, 2002). It is possible that under *in vitro* conditions of our susceptibility tests, gene *tet*(34) was not expressed. Moreover, to date the clinical relevance of gene *tet*(34) is still unknown (Grossman, 2016). In six out of 46 environmental and three out of eight fully sequenced clinical isolates, the *catB9* gene was present. This gene, a chromosome-encoded variant of the *cat* gene found in *V. cholerae* encodes for a chloramphenicol acetyltransferase and confers resistance to chloramphenicol (Rowe-Magnus et al., 2002). In contrast, none of the isolates was resistant to chloramphenicol. No gene conferring sulfonamide resistance (e.g., *sul2*) and streptomycin (e.g., *strB*) for *V. cholerae* was found using either CARD or ResFinder databases, although nearly all of our strains were tested phenotypically resistant to these two antibiotics. These findings indicate that current databases are partly incomplete for assessing antimicrobial resistance properties of *V. cholerae* non-O1/non-O139 strains on genotypic characterization alone. Despite the fact that WGS allows detection of a much higher number of resistance markers, the clinical relevance of most of them still needs to be determined. Clinicians should thus still rely on phenotypic characterization of antimicrobial resistance properties of *V. cholerae* strains instead of depending on *in silico* assessment alone (Nahar and Bin Rashid, 2017). Vice-versa, when a resistance mechanism is detected in the genome, while the isolate is phenotypically susceptible, the interpretation criteria of the antibiogram may also be questioned. In fact, the literature comparing phenotypic and genotypic antimicrobial resistance in *V. cholerae* non-O1/non-O139 is still poor compared to either *E. coli*, *K. pneumoniae* or *S. aureus.* Continuous collection of both data over time may solve such discrepancies between genotype and phenotype in the future.

### Both Clinical and Environmental Isolates Do Not Harbor Integrons

Despite the fact that *V. cholerae* is a highly diverse species that is able to easily take up extracellular DNA (Matthey and Blokesch, 2016), no common integrons and other integrative and conjugative elements able to carry antimicrobial resistance determinants have been identified. All sequenced clinical and environmental strains were negative for SXT elements or class I integron. This finding is in agreement with the other studies (Bier et al., 2015; Ceccarelli et al., 2015; Baron et al., 2017) and suggests that in non-cholera epidemic regions, the dissemination of antibiotic resistance genes via mobile genetic elements through *V. cholerae* colonization of the aquatic environment is probably of very limited impact.

### Ampicillin Resistance Is Concentrated in Specific Phylogenetic Lineages

Finally, we tried to elucidate whether the observed phenotypic and genotypic profiles of the environmental strains were reflected by their phylogeny (see Supplementary Figure S1; Pretzer et al., 2017). Only two resistance traits were suitable for such inter-comparison - ampicillin resistance (*n* = 20) and the presence of the *catB9* gene (*n* = 6), as all other traits were too abundant. From the identified 150 sequence types (STs) (Pretzer et al., 2017), the 20 ampicillin resistant isolates belonged to 15 different STs with eleven of the isolates belonging to only two out of 24 identified phylogenetic clades (Supplementary Table S5). Four isolates belonged to ST 75 (clade III) and three to ST 59 (clade I). In addition, one ST 62 and one ST 65 isolate clustered with ST 75 isolates in clade III and one ST 64 and one ST 76 isolate clustered with ST 59 isolates in clade I. These data show that ampicillin resistance, and consequently the CARB-7 and CARB-9 encoding genes are present only in specific phylogenetic lineages and not evenly distributed among the *V. cholerae* population in the lake. No such phylogenic relationship was observed for the *catB9* gene.

## Conclusion

The large Austrian lake Neusiedler See is an important bathing water with several hundred thousand visitors each year. As it is harboring an abundant and diverse natural *V. cholerae* non-O1/non-O139 community associated with several cases of human infection each year, the comprehensive assessment of the antimicrobial resistance properties of this pathogen is of public health concern. For this purpose, we combined phenotypic antibiotic susceptibility tests with genotypic characterization of environmental and clinical whole-genome-sequenced isolates, covering most of phylogenetic *V. cholerae* diversity in the lake. The specific model characteristics of the lake as a bioreactor for the appearance of new pathogenic/resistant *Vibrio* strains render our findings relevant also for other subsaline/hyposaline aquatic environments that are used for recreational purposes.

Our results clearly showed that all isolates were susceptible to all important first-line and last-line treatment options. Widespread resistance was only observed against old antibiotics such as sulfonamides, streptomycin and ampicillin. The apparent lack of either known antimicrobial resistance traits or mobile genetic elements indicates that in cholera non-epidemic regions of the world, *V. cholerae* non-O1/non-O139 play only a minor role as reservoir of antibiotic resistance genes. Nonetheless, the observed discrepancies between the phenotypic and genotypic resistance data highlight the need for improvement of the resistance databases for *V. cholerae* non-O1/non-O139 to better understand how these pathogens acquire and disseminate resistance traits in the aquatic environment.

## Data Availability Statement

The whole genome sequencing datasets generated for this study can be found in the DDBJ/EMBL/GenBank; accession PRJNA551929.

## Author Contributions

AK, AI, SP, SB, RM, and WR: conceptualization. CP, EL, SB, SL, and SG: methodology. SL, WR, CP, SB, and SG: software analysis. AK, AI, SP, and SB: validation. CP, EL, SB, and SL: investigation. AK, AI, and SB: resources. AK and WR: data curation. SL, SP, SB, AK, CP, EL, SG, and WR: writing–original draft preparation. AK, SB, AI, AF, SP, SG, WR, and RM: writing–review and editing. AK: project administration. AK, AF, AI, and SB: funding acquisition.

## Conflict of Interest

The authors declare that the research was conducted in the absence of any commercial or financial relationships that could be construed as a potential conflict of interest.

## Footnotes

1 http://www.genomicepidemiology.org

2 https://www.ncbi.nlm.nih.gov/bioproject/PRJNA551929/
