# Supplementary material for: Phenotypic and Genotypic Antimicrobial Resistance Traits of Vibrio cholerae Non-O1/Non-O139 Isolated From a Large Austrian Lake Frequently Associated With Cases of Human Infection
Source: Front Microbiol. 2019 Nov 8;10:2600. doi: 10.3389/fmicb.2019.02600 (PMC6857200; doi:10.3389/fmicb.2019.02600)
Supplement: Supplementary file 1 [file Table_1.docx]

| ID | Origin | Year | Specimen | Clinical Symptoms |
| --- | --- | --- | --- | --- |
| CHT 85-09 (VC83) | Neusiedlersee/Podersdorf | 2009 | Ear | Otitis externa |
| P2-CHT15-00 | Neusiedlersee | 2000 | Ear | Otitis externa |
| P7-CHT61-04 | Neusiedlersee | 2004 | Ear | Otitis externa |
| P9-CHT63-05 | Neusiedlersee | 2005 | Blood | Sepsis |
| P10-CHT64-05 | Neusiedlersee | 2005 | Throat Swab | No clinical data available |
| P12-CHT68-05 | Neusiedlersee/Podersdorf | 2005 | Ear | Otitis externa |
| P19-CHT78-07 | Neusiedlersee | 2007 | Ear | Otitis externa |
| 920006-15 | Neusiedlersee | 2015 | Ear | Otitis externa |
| 920008-15 | Neusiedlersee/Mörbisch | 2015 | Urine | Prostatitis |

**Lepuschitz S, Baron S, Larvor E, Granier SA, Pretzer C, Mach RL, Farnleitner AH, Ruppitsch W, Pleininger S, Indra A, Kirschner AKT:** Phenotypic and genotypic antimicrobial resistance traits of *Vibrio cholerae* non-O1/non-O139 isolated from a large Austrian lake frequently associated with cases of human infection

**Supplemental Information - Table S1**: Origin (lake/bathing site, year, specimen) and clinical symptoms of the analysed clinical strains.
