# Supplementary material for: Phenotypic and Genotypic Antimicrobial Resistance Traits of Vibrio cholerae Non-O1/Non-O139 Isolated From a Large Austrian Lake Frequently Associated With Cases of Human Infection
Source: Front Microbiol. 2019 Nov 8;10:2600. doi: 10.3389/fmicb.2019.02600 (PMC6857200; doi:10.3389/fmicb.2019.02600)
Supplement: Supplementary file 2 [file Table_2.docx]

**Lepuschitz S, Baron S, Larvor E, Granier SA, Pretzer C, Mach RL, Farnleitner AH, Ruppitsch W, Pleininger S, Indra A, Kirschner AKT:** Phenotypic and genotypic antimicrobial resistance traits of *Vibrio cholerae* non-O1/non-O139 isolated from a large Austrian lake frequently associated with cases of human infection

**Table S2:** Interpretative criteria used to determine antimicrobial susceptibility with the disk diffusion test in *Vibrio cholerae* isolates. Interpretative criteria specific for *Vibrio* *spp*., including *V. cholerae* described in CLSI document M45 3^rd^ edition (CLSI, 2015). For streptomycin, norfloxacin and nalidixic acid, breakpoints described for *Enterobacteriaceae* in M100 28S (CLSI, 2018) were used. No breakpoints were available for erythromycin. Based on the distribution of the diameter of the inhibition zone only one population could be observed. All the strains were considered as susceptible (Baron et al., 2016).

| Antimicrobial class | Antimicrobial agent | Disk content (µg) | Zone diameter interpretative criteria (mm) | | | Reference |
| --- | --- | --- | --- | --- | --- | --- |
|  |  |  | susceptible | intermediate | resistant |  |
| ß-lactams | Ampicillin | 10 | ≥17 | 14-16 | ≤13 | (CLSI, 2015) |
|  | Amoxicillin-clavulanic acid | 20/10 | ≥18 | 14-17 | ≤13 | (CLSI, 2015) |
|  | Cefotaxime | 30 | ≥26 | 23-25 | ≤22 | (CLSI, 2015) |
|  | Imipenem | 10 | ≥23 | 20-22 | ≤19 | (CLSI, 2015) |
| Phenicols | Chloramphenicol | 30 | ≥18 | 13-17 | ≤12 | (CLSI, 2015) |
| Aminoglycosides | Amikacin | 30 | ≥17 | 15-16 | ≤14 | (CLSI, 2015) |
|  | Gentamicin | 10 | ≥15 | 13-14 | ≤12 | (CLSI, 2015) |
|  | Streptomycin | 10 | ≥15 | 12-14 | ≤11 | (CLSI, 2018) |
|  | Ciprofloxacin | 5 | ≥21 | 16-20 | ≤15 | (CLSI, 2015) |
|  | Nalidixic acid | 30 | ≥19 | 14-18 | ≤13 | (CLSI, 2018) |
|  | Norfloxacin | 10 | ≥17 | 13-16 | ≤12 | (CLSI, 2018) |
| Folate pathway inhibitors | Sulfonamide | 300 | ≥17 | 13-16 | ≤12 | (CLSI, 2015) |
|  | Trimethoprim-sulfamethoxazole | 1.25/23.75 | ≥16 | 11-15 | ≤10 | (CLSI, 2015) |
|  | trimethoprim | 5 | ≥16 | 11-15 | ≤10 | (CLSI, 2018) |
| Tetracyclines | Tetracycline | 30 | ≥15 | 12-14 | ≤11 | (CLSI, 2015) |
| Macrolides | Erythromycin | 15 | For details see manuscript | | | |

C.L.S.I. (2015). Methods for Antimicrobial Dilution and Disk Susceptibility Testing of Infrequently Isolated or Fastidious Bacteria 3rd ed. CLSI guideline. Wayne, PA: Clinical and Laboratory Standards Institute.

C.L.S.I. (2018). "Performance standards for antimicrobial disk suseptibility tests; 13th edition; standard M02". (Wayne, PA, USA: CLSI).

Baron, S., Larvor, E., Chevalier, S., Jouy, E., Kempf, I., Granier, S.A., and Lesne, J. (2017). Antimicrobial Susceptibility among Urban Wastewater and Wild Shellfish Isolates of Non-O1/Non-O139 Vibrio cholerae from La Rance Estuary (Brittany, France). Front Microbiol 8, 1637.
