# Supplementary material for: Phenotypic and Genotypic Antimicrobial Resistance Traits of Vibrio cholerae Non-O1/Non-O139 Isolated From a Large Austrian Lake Frequently Associated With Cases of Human Infection
Source: Front Microbiol. 2019 Nov 8;10:2600. doi: 10.3389/fmicb.2019.02600 (PMC6857200; doi:10.3389/fmicb.2019.02600)
Supplement: Supplementary file 3 [file Table_3.docx]

**Lepuschitz S, Baron S, Larvor E, Granier SA, Pretzer C, Mach RL, Farnleitner AH, Ruppitsch W, Pleininger S, Indra A, Kirschner AKT:** Phenotypic and genotypic antimicrobial resistance traits of *Vibrio cholerae* non-O1/non-O139 isolated from a large Austrian lake frequently associated with cases of human infection

**Supplemental Information - Table S3**: WGS assembly, accession, and BioSample numbers for the strains sequenced in this study.

| **Assembly** | **WGS accession** | **BioSample** | **Strain** | **Taxonomy** |
| --- | --- | --- | --- | --- |
| GCA_006802045.1 | VIOJ00000000 | SAMN12169094 | 920006-15 | Vibrio cholerae |
| GCA_006803115.1 | VIQI00000000 | SAMN12168574 | A110523Z4 | Vibrio cholerae |
| GCA_006803155.1 | VIQJ00000000 | SAMN12168573 | A110509W1 | Vibrio cholerae |
| GCA_006803135.1 | VIQF00000000 | SAMN12168576 | A110829Z5 | Vibrio cholerae |
| GCA_006803125.1 | VIQH00000000 | SAMN12168575 | A110621W3 | Vibrio cholerae |
| GCA_006803105.1 | VIQG00000000 | SAMN12168577 | A110704W4 | Vibrio cholerae |
| GCA_006803045.1 | VIQD00000000 | SAMN12168579 | A110912Z1 | Vibrio cholerae |
| GCA_006803005.1 | VIQB00000000 | SAMN12168581 | A110926W4 | Vibrio cholerae |
| GCA_006803035.1 | VIQE00000000 | SAMN12168578 | A110829Z6 | Vibrio cholerae |
| GCA_006803025.1 | VIQC00000000 | SAMN12168580 | A110912Z3 | Vibrio cholerae |
| GCA_006803015.1 | VIQA00000000 | SAMN12168582 | A111010Z3 | Vibrio cholerae |
| GCA_006802945.1 | VIPZ00000000 | SAMN12168583 | A120416W2 | Vibrio cholerae |
| GCA_006802935.1 | VIPY00000000 | SAMN12168584 | A120502W2 | Vibrio cholerae |
| GCA_006802915.1 | VIPV00000000 | SAMN12168587 | A120618Z1 | Vibrio cholerae |
| GCA_006802905.1 | VIPX00000000 | SAMN12168586 | A120502Z1 | Vibrio cholerae |
| GCA_006802685.1 | VIPM00000000 | SAMN12168607 | A12JL4W72 | Vibrio cholerae |
| GCA_006802925.1 | VIPW00000000 | SAMN12168585 | A120502Z5 | Vibrio cholerae |
| GCA_006802815.1 | VIPS00000000 | SAMN12168599 | A121001W1 | Vibrio cholerae |
| GCA_006802865.1 | VIPU00000000 | SAMN12168588 | A120716Z3 | Vibrio cholerae |
| GCA_006802795.1 | VIPQ00000000 | SAMN12168602 | A12JL4W4 | Vibrio cholerae |
| GCA_006802805.1 | VIPT00000000 | SAMN12168589 | A120730Z1 | Vibrio cholerae |
| GCA_006802785.1 | VIPR00000000 | SAMN12168601 | A121001W2 | Vibrio cholerae |
| GCA_006802705.1 | VIPL00000000 | SAMN12168608 | A12JL4W81 | Vibrio cholerae |
| GCA_006802735.1 | VIPP00000000 | SAMN12168603 | A12JL4W15 | Vibrio cholerae |
| GCA_006802715.1 | VIPO00000000 | SAMN12168604 | A12JL4W21 | Vibrio cholerae |
| GCA_006802695.1 | VIPN00000000 | SAMN12168605 | A12JL4W64 | Vibrio cholerae |
| GCA_006802605.1 | VIPI00000000 | SAMN12168610 | A12JL5W14 | Vibrio cholerae |
| GCA_006802655.1 | VIPJ00000000 | SAMN12168611 | A12JL5W13 | Vibrio cholerae |
| GCA_006802595.1 | VIPH00000000 | SAMN12168612 | A12JL5W24 | Vibrio cholerae |
| GCA_006802635.1 | VIPK00000000 | SAMN12168609 | A12JL4W93 | Vibrio cholerae |
| GCA_006802575.1 | VIPG00000000 | SAMN12168613 | A12JL5W63 | Vibrio cholerae |
| GCA_006802555.1 | VIPF00000000 | SAMN12168615 | A12JL5W86 | Vibrio cholerae |
| GCA_006802545.1 | VIPE00000000 | SAMN12168616 | A12JL5W90 | Vibrio cholerae |
| GCA_006802465.1 | VIPA00000000 | SAMN12168621 | A12JL36W25 | Vibrio cholerae |
| GCA_006802495.1 | VIPB00000000 | SAMN12168619 | A12JL36W17 | Vibrio cholerae |
| GCA_006802475.1 | VIPC00000000 | SAMN12168618 | A12JL36W5 | Vibrio cholerae |
| GCA_006802445.1 | VIOZ00000000 | SAMN12168623 | A12JL36W30 | Vibrio cholerae |
| GCA_006802485.1 | VIPD00000000 | SAMN12168617 | A12JL5W97 | Vibrio cholerae |
| GCA_006802395.1 | VIOX00000000 | SAMN12168624 | A12JL36W52 | Vibrio cholerae |
| GCA_006802375.1 | VIOY00000000 | SAMN12168622 | A12JL36W49 | Vibrio cholerae |
| GCA_006802355.1 | VIOV00000000 | SAMN12168981 | A12JL36W74 | Vibrio cholerae |
| GCA_006802365.1 | VIOW00000000 | SAMN12168980 | A12JL36W67 | Vibrio cholerae |
| GCA_006802295.1 | VIOR00000000 | SAMN12168986 | A12JL36W91 | Vibrio cholerae |
| GCA_006802345.1 | VIOU00000000 | SAMN12168983 | A12JL36W75 | Vibrio cholerae |
| GCA_006802255.1 | VIOQ00000000 | SAMN12168987 | A12JL36W92 | Vibrio cholerae |
| GCA_006802285.1 | VIOS00000000 | SAMN12168985 | A12JL36W90 | Vibrio cholerae |
| GCA_006802275.1 | VIOT00000000 | SAMN12168984 | A12JL36W82 | Vibrio cholerae |
| GCA_006802245.1 | VIOP00000000 | SAMN12169086 | P2-CHT15-00 | Vibrio cholerae |
| GCA_006802205.1 | VION00000000 | SAMN12169088 | P9-CHT63-05 | Vibrio cholerae |
| GCA_006802165.1 | VIOK00000000 | SAMN12169092 | P19-CHT78-07 | Vibrio cholerae |
| GCA_006802195.1 | VIOM00000000 | SAMN12169089 | P10-CHT64-05 | Vibrio cholerae |
| GCA_006802155.1 | VIOO00000000 | SAMN12169087 | P7-CHT61-04 | Vibrio cholerae |
| GCA_006802145.1 | VIOL00000000 | SAMN12169090 | P12-CHT68-05 | Vibrio cholerae |
| GCA_006802075.1 | VIOI00000000 | SAMN12169095 | 920008-15 | Vibrio cholerae |
