# Supplementary material for: Phenotypic and Genotypic Antimicrobial Resistance Traits of Vibrio cholerae Non-O1/Non-O139 Isolated From a Large Austrian Lake Frequently Associated With Cases of Human Infection
Source: Front Microbiol. 2019 Nov 8;10:2600. doi: 10.3389/fmicb.2019.02600 (PMC6857200; doi:10.3389/fmicb.2019.02600)
Supplement: Supplementary file 5 [file Table_5.docx]

**Lepuschitz S, Baron S, Larvor E, Granier SA, Pretzer C, Mach RL, Farnleitner AH, Ruppitsch W, Pleininger S, Indra A, Kirschner AKT:** Phenotypic and genotypic antimicrobial resistance traits of *Vibrio cholerae* non-O1/non-O139 isolated from a large Austrian lake frequently associated with cases of human infection

**Table S5**: Assignment of the ampicillin resistant strains to sequence types and phylogenetic clades. 11 of the 20 ampicillin resistant strains belonged to clade I und III.

| **isolate ID** | **Sequence type** | **Clade** |
| --- | --- | --- |
| A110523W4 | 148 | no clade |
| A110704W3 | 75 | III |
| A110829W4 | 136 | no clade |
| A110829Z3 | 134 | no clade |
| A110926W4 | 130 | XXII |
| A120502Z2 | 123 | no clade |
| A120730Z1 | 75 | III |
| A12JL4W15 | 64 | I |
| A12JL4W21 | 75 | III |
| A12JL5W1 | 76 | I |
| A12JL5W5 | 59 | I |
| A12JL5W24 | 75 | III |
| A12JL5W90 | 59 | I |
| A12JL5W95 | 62 | III |
| A12JL5W100 | 59 | I |
| A12JL36W30 | 105 | no clade |
| A12JL36W49 | 101 | no clade |
| A12JL36W56 | 98 | no clade |
| A12JL36W59 | 65 | III |
| A12JL36W67 | 95 | VIII |
