# Supplementary material for: Phenotypic and Genotypic Antimicrobial Resistance Traits of Vibrio cholerae Non-O1/Non-O139 Isolated From a Large Austrian Lake Frequently Associated With Cases of Human Infection
Source: Front Microbiol. 2019 Nov 8;10:2600. doi: 10.3389/fmicb.2019.02600 (PMC6857200; doi:10.3389/fmicb.2019.02600)
Supplement: Supplementary file 6 [file Data_Sheet_1.pdf]

Lepuschitz S, Baron S, Larvor E, Granier SA, Pretzer C, Mach RL, Farnleitner AH, Ruppitsch W, Pleininger S, Indra A, Kirschner AKT: Phenotypic and genotypic antimicrobial resistance traits of *Vibrio cholerae* non-O1/non-O139 isolated from a large Austrian lake frequently associated with cases of human infection

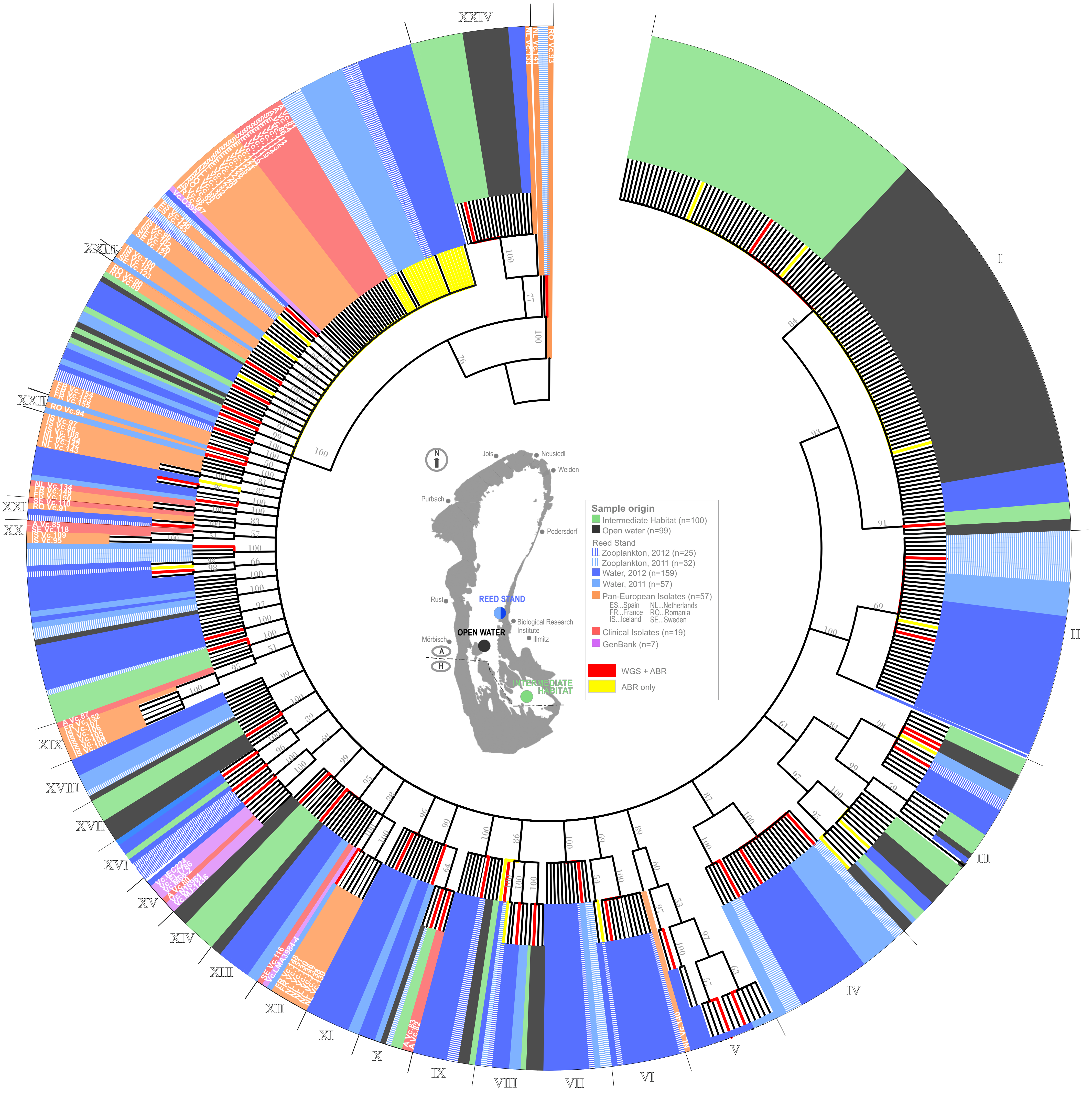

Figure S1: Phylogenetic tree of environmental isolates from Neusiedler See and reference strains, based on multi-locus sequence analysis. Red (Phenotypic antibiotic resistance testing (ABR) and whole genome sequencing (WGS)) and yellow bars (phenotypic antibiotic resistance testing only) indicate the selected isolates analysed in this study. Figure modified after Pretzer et al 2017.

Reference cited:

Pretzer, C., Druzhinina, I. S., Amaro, C., Benediktsdóttir, E., Hedenström, I., Hervio-Heath, D., et al. (2017). High genetic diversity of *Vibrio cholerae* in the European lake Neusiedler see is associated with intensive recombination in the reed habitat and the long-distance transfer of strains. Environ. Microbiol. 19, 328 -344. doi: 10.1111/1462-2920.13612
